# Intermittent Hypoxia and Hypercapnia, a Hallmark of Obstructive Sleep Apnea, Alters the Gut Microbiome and Metabolome

**DOI:** 10.1128/mSystems.00020-18

**Published:** 2018-06-05

**Authors:** Anupriya Tripathi, Alexey V. Melnik, Jin Xue, Orit Poulsen, Michael J. Meehan, Gregory Humphrey, Lingjing Jiang, Gail Ackermann, Daniel McDonald, Dan Zhou, Rob Knight, Pieter C. Dorrestein, Gabriel G. Haddad

**Affiliations:** a Division of Biological Sciences, University of California, San Diego, San Diego, California, USA; b Department of Pediatrics, University of California, San Diego, San Diego, California, USA; c Skaggs School of Pharmacy and Pharmaceutical Sciences, University of California, San Diego, San Diego, California, USA; d Department of Family Medicine and Public Health, University of California, San Diego, San Diego, California, USA; e Department of Computer Science and Engineering, University of California, San Diego, San Diego, California, USA; f Center for Microbiome Innovation, University of California, San Diego, San Diego, California, USA; g Center for Computational Mass Spectrometry, University of California, San Diego, San Diego, California, USA; h Department of Neurosciences, University of California, San Diego, San Diego, California, USA; i Rady’s Children’s Hospital, San Diego, California, USA; Pacific Northwest National Laboratory

**Keywords:** cardiovascular, metabolism, microbiome, sleep apnea

## Abstract

Obstructive sleep apnea (OSA) is a common disorder characterized by episodic obstruction to breathing due to upper airway collapse during sleep. Because of the episodic airway obstruction, intermittently low O_2_ (hypoxia) and high CO_2_ (hypercapnia) ensue. OSA has been associated with adverse cardiovascular and metabolic outcomes, although data regarding potential causal pathways are still evolving. As changes in inspired O_2_ and CO_2_ can affect the ecology of the gut microbiota and the microbiota has been shown to contribute to various cardiometabolic disorders, we hypothesized that OSA alters the gut ecosystem, which, in turn, exacerbates the downstream physiological consequences. Here, we model human OSA and its cardiovascular consequence using *Ldlr*^−/−^ mice fed a high-fat diet and exposed to intermittent hypoxia and hypercapnia (IHH). The gut microbiome and metabolome were characterized longitudinally (using 16S rRNA amplicon sequencing and untargeted liquid chromatography-tandem mass spectrometry [LC-MS/MS]) and seen to covary during IHH. Joint analysis of microbiome and metabolome data revealed marked compositional changes in both microbial (>10%, most remarkably in *Clostridia*) and molecular (>22%) species in the gut. Moreover, molecules that altered in abundance included microbe-dependent bile acids, enterolignans, and fatty acids, highlighting the impact of IHH on host-commensal organism cometabolism in the gut. Thus, we present the first evidence that IHH perturbs the gut microbiome functionally, setting the stage for understanding its involvement in cardiometabolic disorders.

**IMPORTANCE** Intestinal dysbiosis mediates various cardiovascular diseases comorbid with OSA. To understand the role of dysbiosis in cardiovascular and metabolic disease caused by OSA, we systematically study the effect of intermittent hypoxic/hypercapnic stress (IHH, mimicking OSA) on gut microbes in an animal model. We take advantage of a longitudinal study design and paired omics to investigate the microbial and molecular dynamics in the gut to ascertain the contribution of microbes on intestinal metabolism in IHH. We observe microbe-dependent changes in the gut metabolome that will guide future research on unrecognized mechanistic links between gut microbes and comorbidities of OSA. Additionally, we highlight novel and noninvasive biomarkers for OSA-linked cardiovascular and metabolic disorders.

## Abstract (precis)

Intestinal dysbiosis mediates various cardiovascular diseases comorbid with OSA. To understand the role of dysbiosis in cardiovascular and metabolic disease caused by OSA, we systematically study the effect of intermittent hypoxic/hypercapnic stress (IHH, mimicking OSA) on gut microbes in an animal model. We take advantage of a longitudinal study design and paired omics to investigate the microbial and molecular dynamics in the gut to ascertain the contribution of microbes on intestinal metabolism in IHH. We observe microbe-dependent changes in the gut metabolome that will guide future research on unrecognized mechanistic links between gut microbes and comorbidities of OSA. Additionally, we highlight novel and noninvasive biomarkers for OSA-linked cardiovascular and metabolic disorders.

## OBSERVATION

Obstructive sleep apnea (OSA) afflicts nearly 12% of the adult population in the United States, with an annual cost burden of nearly $149.6 billion, per a recent study commissioned by the American Academy of Sleep Medicine (1). Timely diagnosis and treatment of OSA improves not only sleep and cognitive function but also management of comorbid cardiometabolic diseases (CMDs). Therefore, identifying the mechanisms underlying the downstream consequences of OSA would aide in development of effective treatment modalities, reducing overall health care utilization.

OSA is marked by changes in oxygen and carbon dioxide-inspired concentrations, which impact the gut microbial community (2). Since the gut microbiota plays a key role in the metabolism of dietary precursors, including lipids, cholesterol, and choline, it impacts the cardiometabolic health of the host (3). Gut dysbiosis has already been linked to an array of cardiovascular and metabolic disorders, such as hypertension, type 2 diabetes, hepatic steatosis, and atherosclerosis (4, 5). Additionally, previous work has identified specific gut bacteria to be significantly correlated with plasma cholesterol and apolipoprotein levels (6). Thus, probing this commensal ecosystem may provide a valuable avenue of investigation to understand the mechanisms underlying the cardiovascular consequences of OSA. In this study, we investigated the taxonomic and molecular alterations in the gut microbiome that potentially mediate the interplay between OSA and related CMDs.

We used atherosclerosis-prone (*Ldlr*^−/−^) adult mice fed a high-fat diet (HFD) enriched in cholesterol and milk fat (resembling Western dietary practices) to evaluate atherosclerosis risk in OSA. We previously demonstrated that IHH increases atherosclerosis plaque formation in this model (7). As episodic hypoxia and hypercapnia mimic the changes in blood gases that occur in OSA-driven downstream consequences (8), these mice were exposed to IHH (treatment group; *n* = 8) or air (control group; *n* = 8) and examined longitudinally for 6 weeks (see Fig. S1 in the supplemental material). Fecal samples, representative of the gut ecosystem, were collected at baseline and twice each week thereafter, and the microbiome and metabolome were profiled using 16S rRNA amplicon sequencing and liquid chromatography-tandem mass spectrometry (LC-MS/MS)-based untargeted mass spectrometry, respectively. These data were processed to obtain relative abundances of microbial and molecular species per sample (referred to as “feature tables” henceforth), which were used for comparing mice mimicking humans with OSA and control mice.

10.1128/mSystems.00020-18.1 FIG S1  Schematic illustration of the treatment paradigm and sample collection. Groups of 8-week-old male *Ldlr*^*−/−*^ mice were transferred to the treatment room for 2 weeks of acclimatization with room air (RA) and regular chow (RC) food. At 10 weeks of age, mice were switched to a high-fat diet (HFD) and treated with or without intermittent hypoxia and hypercapnia (IHH). The IHH treatment group received 10 h IHH/day in the light cycle for 6 weeks (the blue line was the O_2_ set point, and the green line was the actual level of O_2_; the red line was the CO_2_ set point, and the light-blue line was the actual level of CO_2_). The control groups remained in room air for the same period. Fecal pellets were collected at baseline and twice per week thereafter and were used for microbiome and metabolome analyses. Download FIG S1, TIF file, 0.3 MB. Copyright © 2018 Tripathi et al. 2018 Tripathi et al. This content is distributed under the terms of the Creative Commons Attribution 4.0 International license.

**FIG 1:**
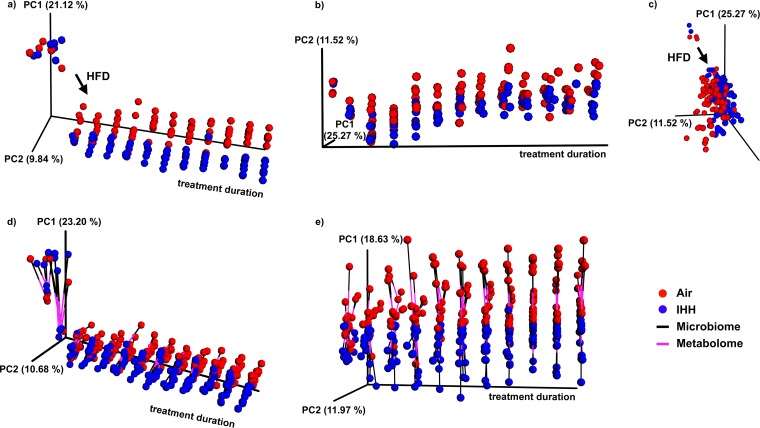
Principal-coordinate analysis (PCoA) and procrustes analysis of the gut microbiome and metabolome. (a) PCoA of the microbiome (16S rRNA sequencing) data using unweighted UniFrac distances; (b and c) PCoA of the metabolome (untargeted LC-MS/MS) data using Gower distances; (d and e) procrustes analysis of the microbiome and metabolome data sets with (d) and without (e) baseline samples. The duration of treatment (in weeks, with an interval length of 0.5 week) is constrained to be one of the axes in the ordination plots (a to e). In the procrustes analysis (d and e), the coordinates for a sample obtained using microbiome data (black lines) are connected to coordinates for the same sample obtained using metabolome data (pink lines). This analysis stretches, rotates, and superimposes ordinations generated from one data set over the other, while preserving distances within each individual matrix. The goal is to find the best fit between two matrices to infer whether one data set coherently captures the properties of the other. PC1, principal component 1; HFD, high-fat diet; IHH, intermittent hypoxia and hypercapnia.

First, we performed principal-coordinate analysis (PCoA) on the microbiome and metabolome feature tables to identify major factors driving the clustering of samples. Figure 1 shows the PCoA results plotted against time to visualize the dynamics of clustering based on the gut microbiome (unweighted UniFrac distances [9] are shown in Fig. 1a) and the metabolome (Gower distances [10] are shown in Fig. 1b and c) as the duration of IHH exposure increases. Here, the first fecal sample represents the baseline gut composition before animals were switched to an HFD. There is a rapid shift in both microbial and molecular composition due to HFD alone, consistent with similar previous findings (11–13). Moreover, starting from a highly congruent gut composition, IHH-exposed mice significantly diverge from controls with increasing exposure duration (a permutational multivariate analysis of variance [PERMANOVA] test was performed per time point [Table S1]). This demonstrates that prolonged IHH exposure (analogous to chronic OSA) cumulatively perturbs the gut microbiome and metabolome. We tested the relationship between the two omics data sets by superimposing the principal coordinates computed from microbiome and metabolome data (procrustes analysis [14]) (Fig. 1d and e). The ordination spaces are correlated (Mantel test *r* statistic = 0.36, *P* < 0.001), and changes in the metabolome and microbiome of samples within the treatment groups over time are proportional, suggesting microbe-dependent changes in intestinal metabolism with chronic OSA.

10.1128/mSystems.00020-18.6 TABLE S1  PERMANOVA results for the gut microbial community (a) and the molecular profile (b) performed for each time point. Download TABLE S1, PDF file, 0.05 MB. Copyright © 2018 Tripathi et al. 2018 Tripathi et al. This content is distributed under the terms of the Creative Commons Attribution 4.0 International license.

**FIG 2:**
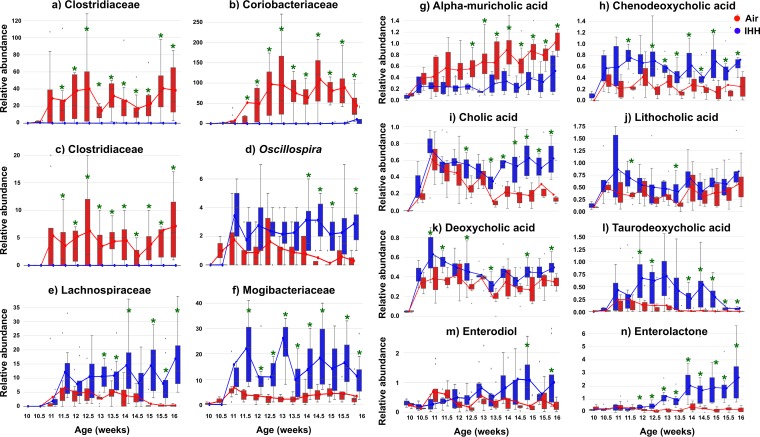
Changes in the gut microbes and molecules due to IHH exposure. (a to f) Top differentially abundant sOTUs elevated in the control group (a, b, and c) and treatment group (d, e, and f). The sOTUs belonging to the families *Clostridiaceae* (a and c) and *Coriobacteriaceae* (b) were elevated in controls, whereas those belonging to the genus *Oscillospira* (d) and the families *Lachnospiraceae* (e) and *Mogibacteriaceae* (f) were higher in IHH-exposed mice than in control mice. (g to n) Trends in abundances of significantly differential bile acids. These differential bile acids include the unconjugated primary bile acids alpha-muricholic acid (g), chenodeoxycholic acid (h), and cholic acid (i), the secondary bile acids lithocholic acid (j) and deoxycholic acid (k), and the conjugated secondary bile acid taurodeoxycholic acid (l). (m and n) Trends in abundances of the significantly differential xenoestrogens enterodiol (m) and enterolactone (n). Significantly differential time points are denoted by asterisks. IHH, intermittent hypoxia and hypercapnia.

We then tested for specific microbes and metabolites that changed with OSA. More than 80 (of ~730) microbial features differed significantly between the IHH-exposed group and controls (by permutation test with discrete false-discovery rate [FDR] correction [15]). Figure S2a presents a global overview of these changes in gut microbiota per sample (sorted by duration of treatment). Table S2 provides a list of these differentially represented bacteria that potentially contribute to alterations in gut metabolism due to IHH. Figure 2a to f display trends in relative abundances of bacteria showing the largest differences, which belong to the *Mogibacteriaceae* (family), *Oscillospira* (genus), *Lachnospiraceae* (family), and *Clostridiaceae* (family). Previous studies have consistently associated these taxonomic groups with metabolic and inflammatory disturbances in the host (16, 17), which suggests that related mechanisms might be at play in driving the consequences of hypoxic and hypercapnic stress.

10.1128/mSystems.00020-18.2 FIG S2  Global overview of changes in the gut microbiota and metabolome. (a) Heatmap of 87 differential microbial sOTUs between IHH-exposed and control mice; (b) heatmap of 382 differential molecular features between IHH-exposed and control mice. Download FIG S2, EPS file, 0.3 MB. Copyright © 2018 Tripathi et al. 2018 Tripathi et al. This content is distributed under the terms of the Creative Commons Attribution 4.0 International license.

10.1128/mSystems.00020-18.7 TABLE S2  List of significantly differential microbial features (uniquely identified by exact 16S sequences). Download TABLE S2, XLS file, 0.1 MB. Copyright © 2018 Tripathi et al. 2018 Tripathi et al. This content is distributed under the terms of the Creative Commons Attribution 4.0 International license.

Using the same statistical approach, we found that more than 380 out of ~1,700 molecular species (MS1 spectral features) differed significantly in relative abundance in animals exposed to IHH. Figure S2b provides a global representation of these differentially abundant molecules in samples belonging to treatment and control groups and sorted by treatment duration (Table S3 provides a comprehensive list of these molecules). To gain insight into the structures of these differentially abundant metabolites, we performed molecular networking using Global Natural Products Social Molecular Networking (GNPS) (18). The molecular network is constructed using a cosine similarity measure between tandem mass spectral data and then visualized using cytoscape (19) (Fig. S3). Each node in the network, which represents a consensus MS/MS spectrum, was searched against public libraries in this study. In total, we annotated about 400 molecular compounds in the GNPS database, including bile acids (BAs), fatty acids, and phytoestrogens. Additionally, all key compounds discussed in this work were defined to the highest level of annotation per the metabolomic standards initiative using commercial standards (Fig. S4, Fig. S5, and Table S4) (20).

10.1128/mSystems.00020-18.3 FIG S3  Molecular network of LC-MS/MS metabolomic data generated with GNPS (rendered using cytoscape 3.4 [19]). Highlighted by boxes are clusters in which differentially abundant metabolites of interest are observed. Color coding of nodes represents metabolite detection in the following groups: IHH only (blue); air only (red); IHH and air (gray); IHH and analytical standard (cyan); air and analytical standard (magenta); IHH, air, and an analytical standard (green); and the standard only (black). Download FIG S3, TIF file, 1.3 MB. Copyright © 2018 Tripathi et al. 2018 Tripathi et al. This content is distributed under the terms of the Creative Commons Attribution 4.0 International license.

10.1128/mSystems.00020-18.4 FIG S4  Comparisons of MS/MS fragmentation spectra. MS/MS fragmentation spectra for annotated molecules are displayed. Fragmentation spectra originating from the most abundant ion is picked for each molecule to display. (Refer to Table S4 for mass/charge and retention time matching to commercial standards.) MS/MS spectra observed in samples and commercial standards are shown on the top and on the bottom for each compound, respectively. Download FIG S4, TIF file, 0.2 MB. Copyright © 2018 Tripathi et al. 2018 Tripathi et al. This content is distributed under the terms of the Creative Commons Attribution 4.0 International license.

10.1128/mSystems.00020-18.5 FIG S5  Comparisons of MS/MS fragmentation spectra. MS/MS fragmentation spectra for annotated molecules are displayed. Fragmentation spectra originating from the most abundant ion is picked for each molecule to display. (Refer to Table S4 for mass/charge and retention time matching to commercial standards.) MS/MS spectra observed in samples and commercial standards are shown on the top and on the bottom for each compound, respectively. Download FIG S5, TIF file, 0.2 MB. Copyright © 2018 Tripathi et al. 2018 Tripathi et al. This content is distributed under the terms of the Creative Commons Attribution 4.0 International license.

10.1128/mSystems.00020-18.8 TABLE S3  List of significantly differential molecular features (uniquely identified as *m/z*_retentionTime). Download TABLE S3, XLS file, 0.1 MB. Copyright © 2018 Tripathi et al. 2018 Tripathi et al. This content is distributed under the terms of the Creative Commons Attribution 4.0 International license.

10.1128/mSystems.00020-18.9 TABLE S4 (a) List of molecular features (named *m/z*_retentionTime) identified using pure analytical standards per the Metabolomics Standards Initiative (L. W. Sumner et al., Metabolomics, 3:211–221, 2007, https://doi.org/10.1007/s11306-007-0082-2). (b). List of pure analytical standards, observed mass spectrometry fragments, and retention times. This information was used for level 1 annotation of molecular features. Download TABLE S4, PDF file, 0.05 MB. Copyright © 2018 Tripathi et al. 2018 Tripathi et al. This content is distributed under the terms of the Creative Commons Attribution 4.0 International license.

Interestingly, the top differentially abundant features detected between IHH and control mice included molecules known to depend on gut microbes for their production. Below, we discuss some of these metabolites and their implications with respect to consequences of OSA.

### Alterations in bile acids.

We observed significant alterations in BAs between IHH-exposed mice and control groups (Table S3). Figure 2g to n display these trends in primary (Fig. 2g to i) and secondary (Fig. 2j to l) bile acids with increasing IHH exposure duration. Primary BAs are amphipathic molecules synthesized in the liver from cholesterol. These are conjugated to glycine or taurine and released in the biliary tract. Together with other biliary components, these facilitate the emulsification and transportation of dietary fats, cholesterol, and fat-soluble vitamins. About 95% of the BAs are reabsorbed in the terminal ileum and recycled. The remaining 5% reach the colon and are deconjugated, dehydrogenated, and dehydroxylated by the intestinal bacteria to form secondary BAs (21). BAs, including microbially generated BAs, are potent signaling molecules that interact with the farnesoid X receptor (FXR) (expressed in the liver and intestine), which modulates BA synthesis by the liver (22). Perturbations in the gut microbial population disrupt normal signaling properties that regulate BA production and can profoundly alter the BA composition in the gut. A range of diseases, including cardiometabolic diseases, are characterized by aberrant BA profiles (23), and prolonged perturbations in the BA pool might also be a factor in mediating the consequences of OSA.

### Elevations in phytoestrogens.

The dietary hormones enterolactone (mammalian lignan) and enterodiol (oxidation product of enterolactone) were significantly elevated in the exposed mice compared to their levels in controls. Figure 2m and n show the trends in their abundances with increasing duration of IHH exposure. These molecules are phytoestrogens, i.e., plant-derived hormones that structurally mimic estrogen and are produced by intestinal microbiota upon bioconversion of dietary lignans. Owing to their affinity to estrogen receptors (producing estrogenic or/and antiestrogenic effects [24]), they perturb many hormone-dependent systems in the body and have been linked to adverse metabolic, reproductive, and neurological outcomes (25). Sex-specific differences in OSA-diagnostic symptoms and risk factors suggest hormonal involvement (26, 27). However, the contribution of microbes in maintaining hormonal homeostasis has not yet been investigated. Therefore, these findings motivate novel avenues of research for biomarkers and therapeutic targets to manage the metabolic consequences of OSA.

### Alterations in fatty acids.

In addition to detecting changes in bile acids and phytoestrogens, we detected differentially abundant fatty acid-related chemical families (Table S3). For example, we noted a significant reduction in a molecular feature matched to elaidic acid. Elaidic acid is an unsaturated fatty acid that increases plasma cholesteryl ester transfer protein (CETP) activity, which modulates systemic levels of low-density lipoprotein (LDL) and high-density lipoprotein (HDL) cholesterol. A decrease in elaidic acid in the IHH-exposed group suggests reduction in plasma CETP activity, a mechanism associated with adverse cardiovascular effects (28). Similarly, phytomonic, jasmonic, hexadecanoic, linoleic, and conjugated linoleic acids were also reduced in exposed mice compared to levels in controls. Of these, phytomonic acid and conjugated linoleic acid are known to be microbially produced (29, 30), suggesting that changes in the microbiome contribute to these changes in metabolome.

In summary, we demonstrate that IHH, a hallmark of OSA, changes the microbiota and the chemistry in the gut. We have highlighted changes in bile acids, phytoestrogens, and fatty acids under OSA-related conditions that could lead to CMDs. The present results reveal a previously unrecognized mechanistic link between OSA and gut microbes. It suggests that targeting gut microbiota and their metabolites may serve as a potential therapeutic approach for the treatment of cardiometabolic consequences of OSA patients.

### Animals.

Atherosclerosis-prone 10-week-old male *Ldlr*^−/−^ mice in the C57BL/6J background (stock number 002207; The Jackson Laboratory, Bar Harbor, ME) were used in this study (31). *Ldlr* deficiency was confirmed by PCR per the vendor’s instructions. All animal protocols were approved by the Animal Care Committee of the University of California, San Diego, and followed the Guide for the Care and Use of Laboratory Animals of the National Institutes of Health (32).

### High-fat diet treatment.

Starting at 10 weeks of age, male mice were provided with a high-fat diet (HFD) containing 1.25% cholesterol and 21% milk fat (4.5 kcal/g; TD96121; Harlan-Teklad, Madison, WI) for 6 weeks while being exposed to either IHH or room air.

### IHH exposure.

Intermittent hypoxia and hypercapnia (IHH) was maintained in a computer-controlled atmosphere chamber system (OxyCycler; Reming Bioinstruments, Redfield, NY) as previously described (7). IHH exposure was introduced to the mice in short periods (~4 min) of synchronized reduction of O_2_ (from 21% to 8%) and elevation of CO_2_ (from ~0.5% to 8%), separated by alternating periods (~4 min) of normoxia ([O_2_] = 21%) and normocapnia ([CO_2_] = ~0.5%), with 1- to 2-min ramp intervals for 10 h per day during the light cycle for 6 weeks. This treatment protocol mimics the severe clinical condition observed in obstructive sleep apnea patients. Mice on the same HFD but in room air were used as controls.

As the experimental setup requires IHH-exposed mice in a controlled atmosphere chamber and controls in room air, we ensured that the effect of treatment is not confounded by the effect of distinct housing conditions. To do so, we used two cages per treatment group, and we compared the relative effect sizes of treatments and cages with redundancy analysis (RDA), which estimates the independent effect size of each covariate on microbiome composition variation based on unweighted UniFrac distance (33). The RDA results showed that treatment had a higher effect size than the cages, more specifically, that treatment contributed to 11.6% of the microbiome community variation, while cages had an independent effect size of around 9.8%; with respect to the metabolome, treatment contributed to 6.2% of the variation, while cages contributed to only about 0.7% of the variation.

### LC-MS/MS data acquisition.

Prior to LC-MS/MS analysis, fecal samples were prepared using the following extraction procedures. For extraction, 500 µl of 50:50 methanol-H_2_O was added to all fecal samples (30 to 50 mg approximately) and vortexed. Fecal pellets in extraction solvent were placed in an ultrasonic bath and sonicated for 30 min to break apart the pellet and then allowed to incubate for an additional 30 min. Extracted samples were then centrifuged to separate insoluble material, and 450 µl of each liquid extract was subsequently transferred to a 96-well deep-well plate and dried completely using centrifugal evaporation (CentriVap centrifugal vacuum concentrator; Labconco, Kansas City, MO). The dried extracts were resuspended in 150 µl of methanol-H_2_O (1:1, vol/vol), with 1 µM amitriptyline included as an autosampler injection standard. After resuspension, the samples were transferred into 96-well plates and analyzed on a Vanquish ultrahigh-performance liquid chromatography (UPLC) system coupled to a Q Exactive orbital ion trap (Thermo Fisher Scientific, Bremen, Germany). For the chromatographic separation, a C_18_ core shell column (Kinetex column, 50 by 2 mm, 1.7-µm particle size, 100-Å pore size; Phenomenex, Torrance, CA) with a flow rate of 0.5 ml/min (solvent A, H_2_O-0.1% formic acid [FA]; solvent B, acetonitrile-0.1% FA) was used. After being injected, the samples were eluted from 0 to 0.5 min with a linear gradient of 5% solvent B, from 0.5 to 4 min with 5 to 50% solvent B, and from 4 to 5 min with 50 to 99% solvent B, followed by a 2-min washout phase at 99% solvent B and a 2-min reequilibration phase at 5% solvent B. For online MS/MS measurements, the flow was directed to a heated electrospray ionization (HESI) source. The ESI parameters were set to 35 liters/min for the sheath gas flow, 10 liter/min for the auxiliary gas flow, 2 liters/min for the sweep gas flow, and 400°C for the auxiliary gas temperature. The spray voltage was set to 3.5 kV, and the inlet capillary was set to 250°C. A 50-V S-lens radio frequency (RF) level was applied. Product ion spectra were recorded in data-dependent acquisition (DDA) mode. Both MS1 survey scans (*m/z* 150 to 1,500) and up to 5 MS/MS scans of the most abundant ions per duty cycle were measured with a resolution (R) of 17,500 with 1 microscan in positive mode. The maximum ion injection time was set to 100 ms. MS/MS precursor selection windows were set to *m/z* 3 with an *m/z* 0.5 offset. Normalized collision energy was stepwise increased from 20 to 30 to 40% with a *z* of 2 as the default charge state. MS/MS experiments were automatically triggered at the apex of a peak within 2 to 15 s from their first occurrence. Dynamic exclusion was set to 5 s.

### LC-MS/MS data analysis.

Feature detection was as follows. Thermo raw data sets were converted to *m/z* extensible markup language (mzXML) in centroid mode using MSConvert (part of ProteoWizard) (34, 35). All mzXML files were cropped with an *m/z* range of 75.00 to 1,000.00 Da. MS1-based feature detection and MS2-based molecular networking was performed using the GNPS workflow (https://gnps.ucsd.edu/ProteoSAFe/static/gnps-splash.jsp) (18). The parameters used are detailed at the following URL: http://gnps.ucsd.edu/ProteoSAFe/status.jsp?task=c6438af750784d919dcd0ee0a783b4fc. Feature extraction parameters were optimized using MZmine2 (http://mzmine.sourceforge.net/) (36) with a signal threshold of 2.0e5 and a 0.3-s minimum peak width. The mass tolerance was set to 10 ppm, and the maximum allowed retention time deviation was set to 10 s. For chromatographic deconvolution, the local minimum search algorithm was used with a minimum relative peak height of 1% and a minimum retention time range of 0.6 s. The maximum peak width was set to 1 min. After isotope peak removal, the peak lists of all samples were aligned with the above-mentioned retention time and mass tolerances. After the creation of a feature matrix containing the feature retention times and the exact mass and peak areas of the corresponding extracted ion chromatograms, the metadata of the samples (treatment type and duration) were added. The signal intensities of the features were normalized (probabilistic quotient normalization [PQN]) (37) to an internal standard (*m/z* 278.189; real time, 3.81 min) for subsequent analysis.

MS/MS annotations were as follows. Molecular features, in the form of MS/MS spectra, were putatively identified using MS2-based spectral library matches. The false-discovery rate (FDR) was estimated using a decoy database approach (38) in GNPS and was found to be less than 1% above a cosine similarity score of 0.6 (GNPS job link, https://gnps.ucsd.edu/ProteoSAFe/status.jsp?task=feac48de4c9f45d485403e3feb7a470d). Therefore, we used a cosine score of 0.65 here. For level 1 annotation, as defined by the 2007 metabolomics standards initiative for differentially abundant metabolites, we purchased authentic standards of alpha-muricholic acid, chenodeoxycholic acid, cholic acid, lithocholic acid, deoxycholic acid, taurodeoxycholic acid, and the xenoestrogens enterodiol and enterolactone from Cayman Chemical (Ann Arbor, MI) and analyzed them using the same LC-MS/MS method described above. We then compared and verified the exact masses, fragmentation patterns, and retention times of those compounds to ensure correct annotations (Fig. S2 and Fig. S3).

Statistical analysis was carried out as follows. QIIME 1.9.1 was used to perform principal-coordinate analysis (PCoA) (beta_diversity.py, a Gower dissimilarity metric [10]) and a PERMANOVA test (compare_categories.py). The PCoA plots were visualized in EMPeror (39). Differential abundance analysis was performed using discrete FDRs (15).

### 16S rRNA sequencing.

DNA extraction and 16S rRNA amplicon sequencing were done using Earth Microbiome Project (EMP) standard protocols (http://www.earthmicrobiome.org/protocols-and-standards/16s) (40). In brief, DNA was extracted using the MO BIO PowerSoil DNA extraction kit (Carlsbad, CA). Amplicon PCR was performed on the V4 region of the 16S rRNA gene using the primer pair 515f to 806r with Golay error-correcting barcodes on the reverse primer. Amplicons were barcoded and pooled in equal concentrations for sequencing. The amplicon pool was purified with the MO BIO UltraClean PCR cleanup kit and sequenced on the Illumina HiSeq 2500 sequencing platform. Sequence data were demultiplexed and minimally quality filtered using the QIIME 1.9.1 script split_libraries_fastq.py, with a Phred quality threshold of 3 and default parameters to generate per-study FASTA sequence files.

### 16S marker gene data analysis.

Feature detection and identification were performed as follows. The raw sequence data were processed using the Deblur workflow (41) with default parameters in Qiita (https://qiita.ucsd.edu/). This generated a sub-operational taxonomic unit (sOTU) abundance per sample (BIOM format) (41, 42). Taxonomies for sOTUs were assigned using the sklearn-based taxonomy classifier (feature classifier plug-in) in QIIME 2 (43). The sOTU table was rarefied to a depth of 2,000 sequences/sample to control for sequencing effort (44). A phylogeny was inferred using SATé-enabled phylogenetic placement (45), which was used to insert 16S Deblur sOTUs into Greengenes 13_8 at a 99% phylogeny.

For statistical analysis, QIIME 2 was used to perform PCoA (unweighted UniFrac distances [9]). QIIME 1.9.1 was used for the PERMANOVA test (compare_categories.py), Mantel test (compare_distance_matrices.py), and procrustes analysis (transform_coordinate_matrices.py). The PCoA and procrustes plots were visualized in EMPeror. (39) Differential-abundance analysis was performed using discrete FDRs (15).

### Data availability.

The data generated in this study are available publicly under the following accession numbers: for metabolomics data, MSV000081482; for commercial standards, MSV000081853; and for microbiome data, ERP106495 (EBI database). Data analysis has been documented in Jupyter notebooks available on GitHub (https://github.com/knightlab-analyses/haddad_osa).
