# Supplementary figures and images for: Intermittent Hypoxia and Hypercapnia, a Hallmark of Obstructive Sleep Apnea, Alters the Gut Microbiome and Metabolome
Source: mSystems. 2018 Jun 5;3(3):e00020-18. doi: 10.1128/mSystems.00020-18 (PMC5989129; doi:10.1128/mSystems.00020-18)

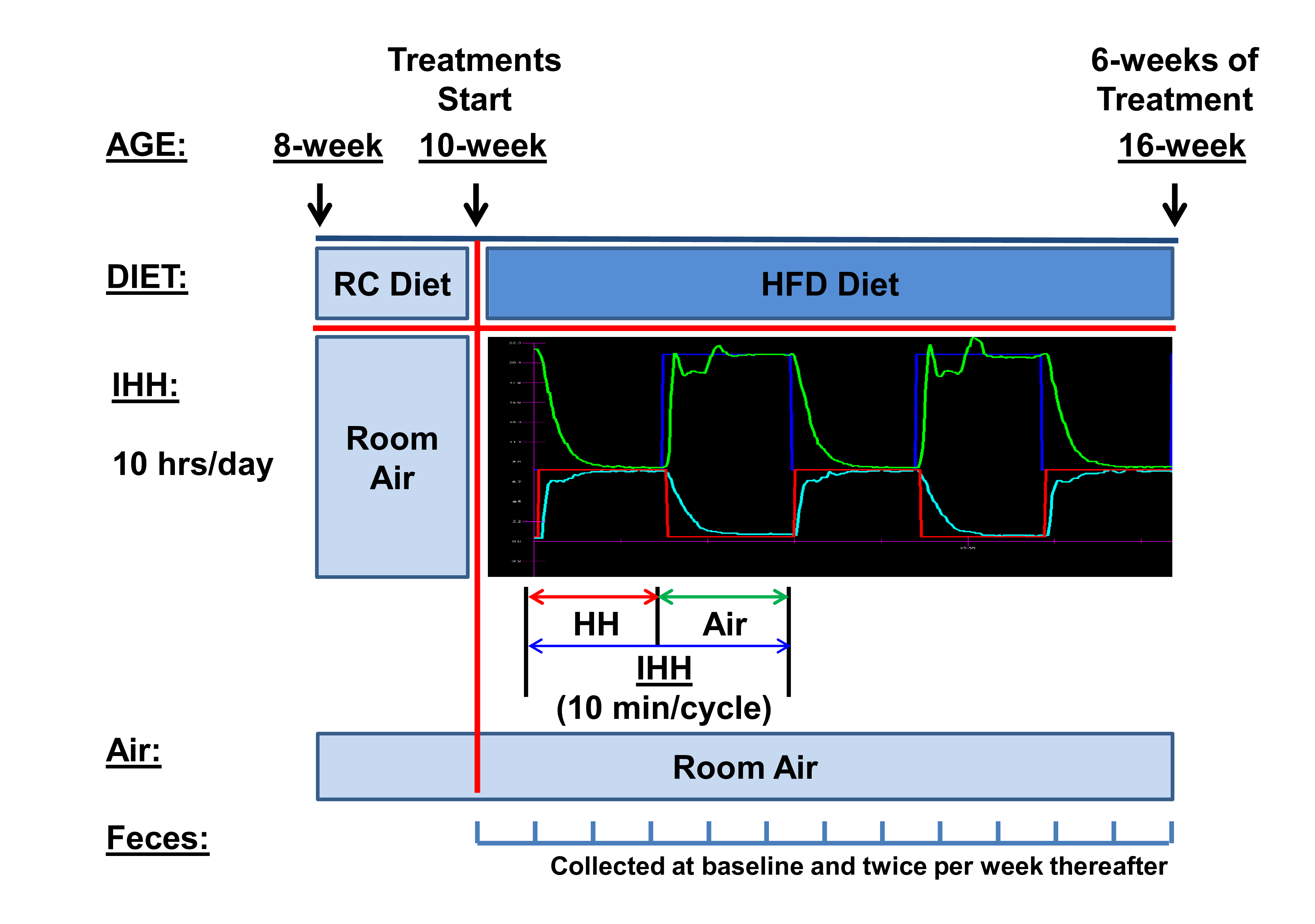

Supplement: FIG S1 [file sys003182235sf1.tif]

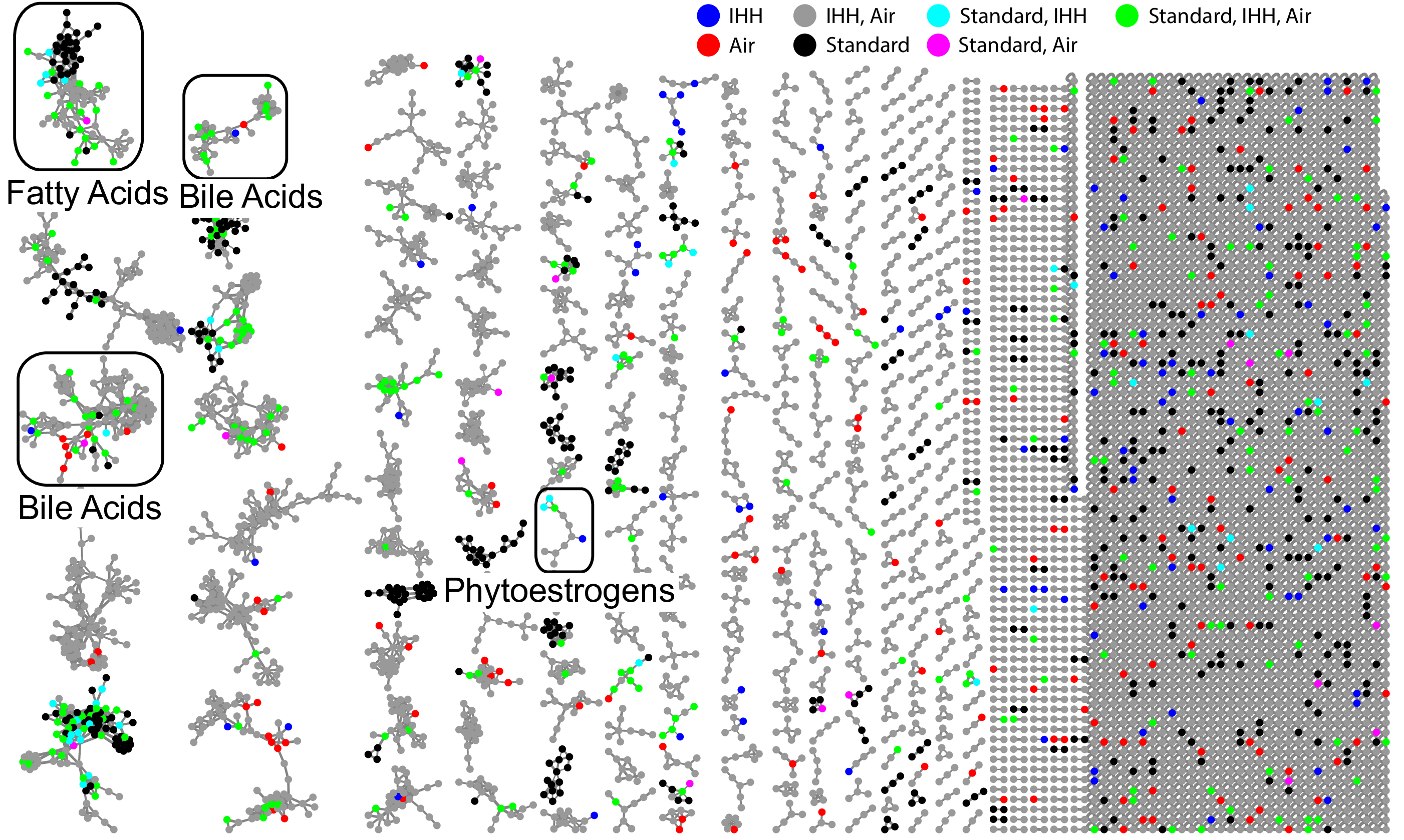

Supplement: FIG S3 [file sys003182235sf3.tif]

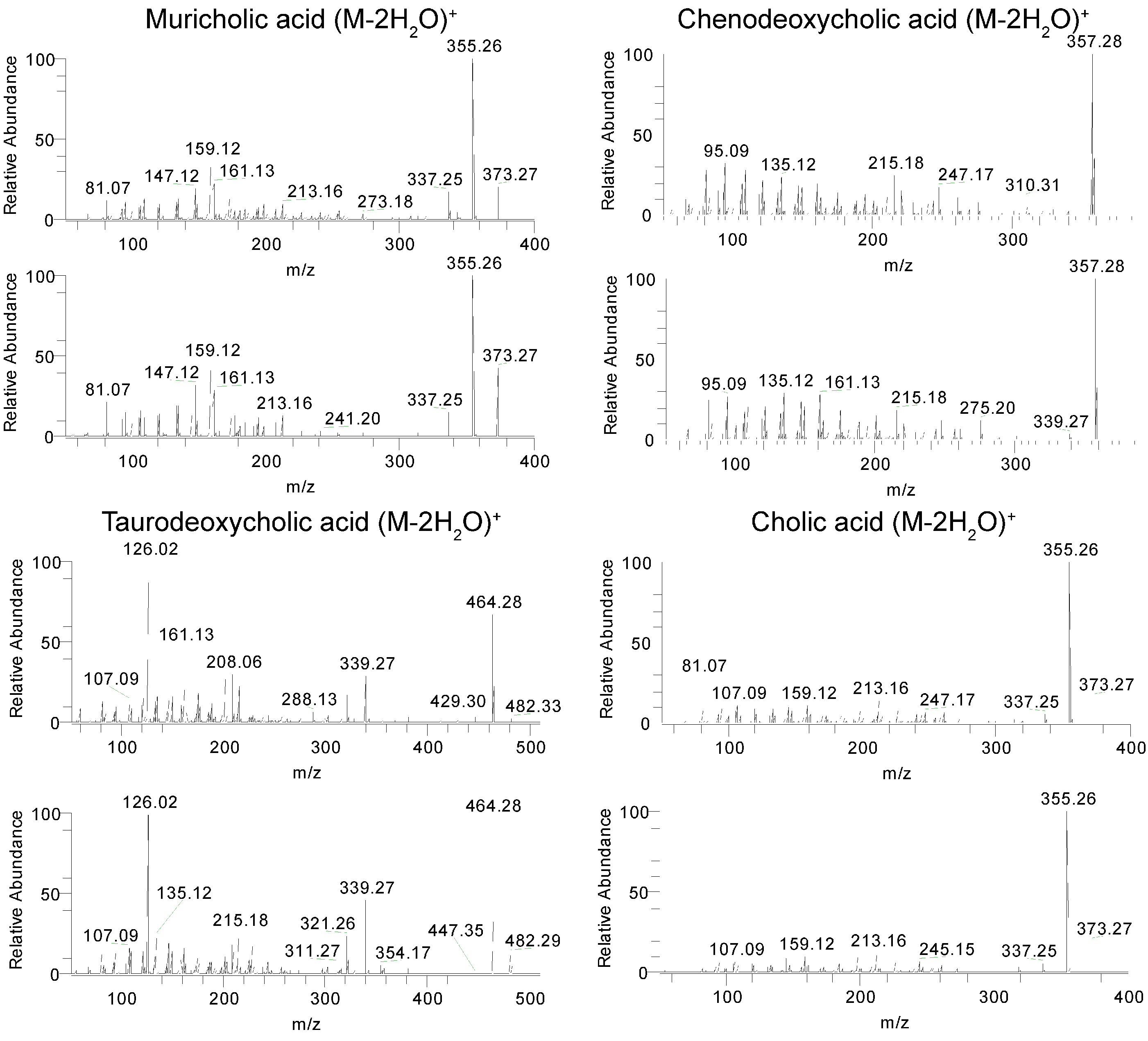

Supplement: FIG S4 [file sys003182235sf4.tif]

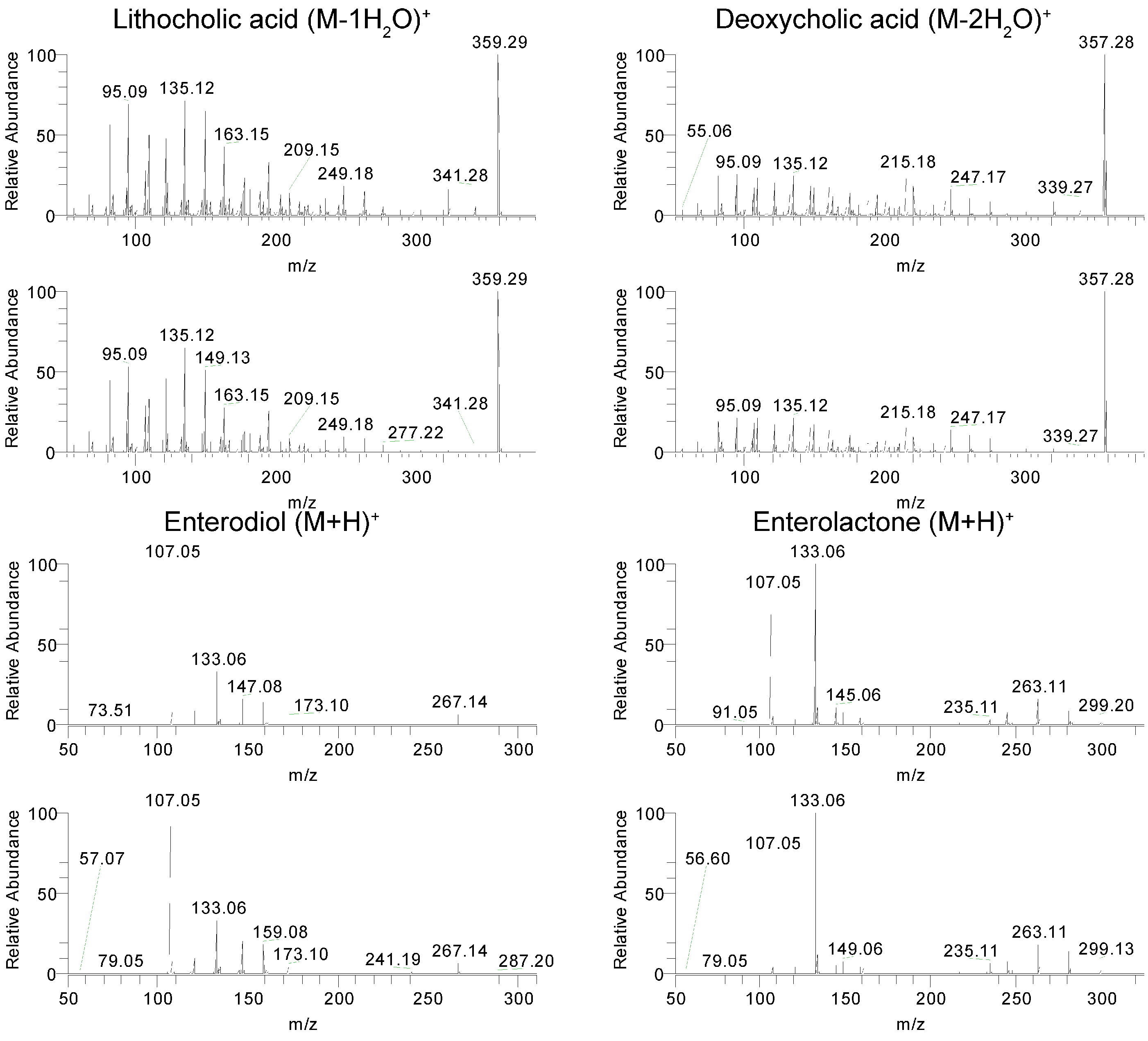

Supplement: FIG S5 [file sys003182235sf5.tif]
