# Supplementary material for: Intermittent Hypoxia and Hypercapnia, a Hallmark of Obstructive Sleep Apnea, Alters the Gut Microbiome and Metabolome
Source: mSystems. 2018 Jun 5;3(3):e00020-18. doi: 10.1128/mSystems.00020-18 (PMC5989129; doi:10.1128/mSystems.00020-18)
Supplement: TABLE S1 [file sys003182235st1.pdf]

Table S1. (a) PERMANOVA analyses of the gut microbial community

Age (weeks)	F statistic	p-value
10	1.276182718	0.212
10.5	0.770229415	0.955
11	1.800742236	0.022
11.5	2.13993268	0.008
12	2.68313051	0.002
12.5	2.273922463	0.005
13	3.494189164	0.001
13.5	3.938096095	0.001
14	3.563734605	0.001
14.5	2.933013157	0.002
15	3.014646002	0.001
15.5	3.721877392	0.001
16	4.056393228	0.001

Table S1. (b) PERMANOVA analyses of the gut molecular profile performed for each time point.

Age (weeks)	F statistic	p-value
10	1.174839941	0.306
10.5	1.193019523	0.308
11	2.114917029	0.037
11.5	3.197963429	0.001
12	2.373602645	0.017
12.5	2.437923273	0.006
13	2.892192261	0.005
13.5	3.938096095	0.001
14	3.563734605	0.001
14.5	2.829598616	0.007
15	2.433892265	0.019
15.5	3.721877392	0.001
16	2.563095343	0.009
