# Supplementary material for: Intermittent Hypoxia and Hypercapnia, a Hallmark of Obstructive Sleep Apnea, Alters the Gut Microbiome and Metabolome
Source: mSystems. 2018 Jun 5;3(3):e00020-18. doi: 10.1128/mSystems.00020-18 (PMC5989129; doi:10.1128/mSystems.00020-18)
Supplement: TABLE S4 [file sys003182235st4.pdf]

Table S4 (a). List of molecular features (named as m/z_retentionTime) identified using pure analytical standards according to Metabolomics Standard Initiative (Sumner, L. W. et al., Metabolomics, 2007)

#featureID	pvalue	level2_gnps	test_statistic	obs_mz	obs_rt	level_1_identification	trend
355.2629055490768_3.7938971153846124	1.00E-04	Spectral Match to Cholic acid from NIST14	-56.24855072	355.262906	3.79389712	a-Muricholic acid	higher in Air
373.27348615624044_3.8406199677938813	1.00E-04	Spectral Match to Cholic acid from NIST14	-45.74178744	373.273486	3.84061997	a-Muricholic acid	higher in Air
391.28410117004233_3.8488245192307704	1.00E-04	Spectral Match to 12-Ketodeoxycholic acid from NIST14	-37.12536232	391.284101	3.84882452	a-Muricholic acid	higher in Air
817.5817207148909_3.8418794166666657	1.00E-04	Spectral Match to Cholic acid from NIST14	-35.31195652	817.581721	3.84187942	a-Muricholic acid	higher in Air
785.5902260763991_4.755478546099291	1.00E-04	Spectral Match to Deoxycholic acid from NIST14	51.10507246	785.590226	4.75547855	Chenodeoxycholic acid	higher in IHH
375.2881148445714_4.7519132211538455	1.00E-04	Spectral Match to Ursodeoxycholic acid from NIST14	52.29202899	375.288115	4.75191322	Chenodeoxycholic acid	higher in IHH
357.2784642647587_4.740846153846155	1.00E-04	ctral Match to 3.beta.-Hydroxy-5-cholenoic acid from NIS	56.82004831	357.278464	4.74084615	Chenodeoxycholic acid	higher in IHH
339.2681037729704_4.738620238095239	1.00E-04	none	61.77669082	339.268104	4.73862024	Chenodeoxycholic acid	higher in IHH
373.27350038542585_4.124289342948718	0.00069993	Spectral Match to Cholic acid from NIST14	25.84927536	373.2735	4.12428934	Cholic acid	higher in IHH
355.2629006371635_4.131933012820513	0.00019998	Spectral Match to Cholic acid from NIST14	26.37681159	355.262901	4.13193301	Cholic acid	higher in IHH
391.2840865306042_4.331681730769233	1.00E-04	Spectral Match to 12-Ketodeoxycholic acid from NIST14	36.4	391.284087	4.33168173	Cholic acid	higher in IHH
355.2629143700245_4.354349038461536	1.00E-04	Spectral Match to Cholic acid from NIST14	37.52101449	355.262914	4.35434904	Cholic acid	higher in IHH
373.27348764725264_4.338406810897439	1.00E-04	Spectral Match to Cholic acid from NIST14	39.21352657	373.273488	4.33840681	Cholic acid	higher in IHH
817.5817957480525_4.338310569105691	1.00E-04	Spectral Match to Cholic acid from NIST14	43.58768116	817.581796	4.33831057	Cholic acid	higher in IHH
391.2841105382007_4.130280368589744	1.00E-04	Spectral Match to 12-Ketodeoxycholic acid from NIST14	48.70917874	391.284111	4.13028037	Cholic acid	higher in IHH
817.5818611613716_4.130555448717949	1.00E-04	Spectral Match to Cholic acid from NIST14	50.35772947	817.581861	4.13055545	Cholic acid	higher in IHH
785.5917246353223_5.009369484702093	1.00E-04	Spectral Match to Deoxycholic acid from NIST14	39.19154589	785.591725	5.00936949	Deoxycholic acid	higher in IHH
375.2884788491843_5.009893639291467	1.00E-04	Spectral Match to Ursodeoxycholic acid from NIST14	40.22463768	375.288479	5.00989364	Deoxycholic acid	higher in IHH
339.268128220786_5.009364251207728	1.00E-04	none	41.80724638	339.268128	5.00936425	Deoxycholic acid	higher in IHH
357.2784138555112_5.010000161030595	1.00E-04	ctral Match to 3.beta.-Hydroxy-5-cholenoic acid from NIS	43.49975845	357.278414	5.01000016	Deoxycholic acid	higher in IHH
285.1479281026352_3.2348193910256406	0.00029997	Spectral Match to Enterodiol from NIST14	30.57512077	285.147928	3.23481939	Enterodiol	higher in IHH
299.12717053502917_3.77541909385113	1.00E-04	Spectral Match to Enterolactone from NIST14	60.77657005	299.127171	3.77541909	Enterolactone	higher in IHH
357.27789601970994_4.411550985221675	0.00169983	ctral Match to 3.beta.-Hydroxy-5-cholenoic acid from NIS	24.20072464	357.277896	4.41155099	Hyodeoxycholic acid	higher in IHH
341.28327251080486_5.417118203883496	1.00E-04	none	37.01545894	341.283273	5.4171182	Lithocholic acid	higher in IHH
359.2929025956458_5.417774879227055	1.00E-04	Lithocholic acid	38.92777778	359.292903	5.41777488	Lithocholic acid	higher in IHH
480.2783022355117_3.0232786991869927	1.00E-04	none	31.03671498	480.278302	3.0232787	Tauro-b-muricholic acid	higher in IHH
498.2882827904067_3.503280892255893	0.00039996	none	29.10241546	498.288283	3.50328089	Taurocholic acid	higher in IHH
480.27763965743867_3.5192286858974375	0.00049995	none	29.12439614	480.27764	3.51922869	Taurocholic acid	higher in IHH
464.2825194019401_3.911678431372549	1.00E-04	none	30.2673913	464.282519	3.91167843	Taurodeoxycholic acid	higher in IHH
482.29143548395945_3.907954655493486	1.00E-04	none	31.22355072	482.291436	3.90795466	Taurodeoxycholic acid	higher in IHH
482.29186826667524_3.770169765494136	1.00E-04	none	51.37983092	482.291868	3.77016977	Taurodeoxycholic acid	higher in IHH
464.2829014176653_3.757069696969693	1.00E-04	none	58.11690821	464.282901	3.7570697	Taurodeoxycholic acid	higher in IHH
375.28808768087396_4.528944230769232	0.00219978	Spectral Match to Ursodeoxycholic acid from NIST14	23.45338164	375.288088	4.52894423	Ursodeoxycholic acid	higher in IHH

Table S4 (b). List of pure analytical standards, observed mass-spectrometry fragments and retention times. This information was used for level 1 annotation of molecular features

Name	Abbreviation	Mol Formula	Theoretical m/z	-1 H_2O	-2 H_2O	-3 H_2O	Dimer	Trimer	RT standard QE	RT sec
Cholic acid	CA	$C_{24}H_{40}O_5$	409.295	391.285	373.275	355.265	817.582	1225.89	4.23	253.8
Deoxycholic acid	DCA	$C_{24}H_{40}O_4$	393.3	375.29	357.28	339.268	785.593	1177.91	4.91	294.6
Glycocholic acid	GCA	$C_{26}H_{43}NO_6$	466.317	448.307	430.297	412.287	931.623	1396.96	3.8	228
Glycochenodeoxycholic acid	GCDCA	$C_{26}H_{43}NO_5$	450.322	432.312	414.302	not obs.	899.635	1348.97	4.31	258.6
Glycolithocholic acid	GLCA	$C_{26}H_{43}NO_4$	434.327	416.317	not obs.	not obs.	867.645	1322.97	4.98	298.8
Hyodeoxycholic acid	HDCA	$C_{24}H_{40}O_4$	393.3	375.29	357.28	339.27	785.591	1177.91	4.29	257.4
Lithocholic acid	LCA	$C_{24}H_{40}O_3$	377.306	359.296	341.286	323.276	753.6	1129.898	5.34	320.4
Taurocholic acid	TCA	$C_{26}H_{45}NO_7S$	516.3	498.289	480.279	462.269	1031.588	1546.91	3.38	202.8
Taurodeoxycholic acid	TDCA	$C_{26}H_{45}NO_6S$	500.305	482.295	464.285	not obs.	999.6	1498.93	3.84	230.4
Taurolithocholic acid	TLCA	$C_{26}H_{45}NO_5S$	484.31	466.3	448.29	not obs.	967.61	1450.94	4.34	260.4
Ursodeoxycholic acid	UDCA	$C_{24}H_{40}O_4$	393.3	375.29	357.28	339.27	785.61	1177.882	4.38	262.8
Chenodeoxycholic acid	CDCA	$C_{24}H_{40}O_4$	393.3	375.29	357.28	339.268	785.593	1177.91	4.87	292.2
b-Muricholic acid	bMCA	$C_{24}H_{40}O_5$	409.295	391.285	373.275	355.265	817.582	1226.39	4.11	246.6
Tauro-b-muricholic acid	TbMCA	$C_{26}H_{45}NO_7S$	516.3	498.289	480.279	462.269	1031.61	1546.912	3.06	183.6
Murideoxycholic acid	MDCA	$C_{24}H_{40}O_4$	393.3	375.29	357.28	339.27	785.591	1177.91	4.26	255.6
a-Muricholic acid	aMCA	$C_{24}H_{40}O_5$	409.295	391.285	373.275	355.265	817.582	1225.89	3.88	232.8
Hyocholic acid; g-Muricholic acid	HCA	$C_{24}H_{40}O_5$	409.295	391.285	373.275	355.265	817.582	1225.89	4.11	246.6
Enterolactone	ENL	$C_{18}H_{18}O_4$	299.127	not obs.	not obs.	not obs.	not obs.	not obs.	3.77	226.2
Enterodiol	END	$C_{18}H_{22}O_4$	285.148	not obs.	not obs.	not obs.	not obs.	not obs.	3.23	193.8
